# Upper-limb rehabilitation interventions delivered by healthcare professionals for adult patients in the intensive care unit setting: protocol for a scoping review

**DOI:** 10.1136/bmjopen-2025-110429

**Published:** 2025-12-03

**Authors:** James Bruce, Ema Swingwood, Sarah Barrett, Emma Dures

**Affiliations:** 1 University Hospitals Bristol and Weston NHS Foundation Trust, Bristol, England, UK; 2 NIHR Bristol Biomedical Research Centre, University of Bristol, Bristol, UK; 3 School of Health and Social Wellbeing, University of the West of England, Bristol, UK

**Keywords:** Protocols & guidelines, Adult intensive & critical care, Patient-Centered Care, REHABILITATION MEDICINE

## Abstract

**Introduction:**

Post-intensive care syndrome affects up to 70% of adult intensive care unit (ICU) survivors, with ICU-acquired weakness contributing substantially to long-term disability. Despite evidence supporting early and structured rehabilitation to enhance physical recovery, targeted upper-limb rehabilitation approaches in the ICU remain comparatively underexplored. This scoping review will map and summarise existing evidence on upper-limb weakness and rehabilitation strategies delivered by healthcare professionals for critically ill adults.

**Methods and analysis:**

This scoping review protocol has been developed in accordance with the Joanna Briggs Institute methodology for scoping reviews and the Preferred Reporting Items for Systematic Reviews and Meta-Analyses extension for Scoping Reviews, ensuring transparent and comprehensive reporting. Searches will be conducted in MEDLINE (Ovid), CINAHL (EBSCO), Embase, Scopus and the Cochrane Central Register of Controlled Trials (CENTRAL). The search will include studies published between March 2009 and August 2025, aligning with National Institute of Clinical Excellence (NICE) Guideline CG83 (2009), which marked a major policy shift in ICU rehabilitation practice. Quantitative data will be summarised descriptively (eg, frequencies and proportions), while qualitative data will undergo thematic synthesis to identify patterns in experiences, perceptions and implementation of upper-limb rehabilitation. Grey literature (eg, OpenGrey and relevant conference proceedings) will also be screened to reduce publication bias. Rayyan AI software will be used to manage citation screening and reviewer collaboration; no artificial intelligence-assisted decision tools will be used to determine study inclusion.

**Ethics and dissemination:**

As this review will synthesise previously published data, ethical approval is not required. Results will be disseminated through peer-reviewed publication, conference presentations and open-access platforms. Findings from this review will inform the development of evidence-based ICU rehabilitation guidelines and highlight priorities for future research to improve upper-limb recovery in critically ill adults.

**Study registration:**

Open Science Framework (osf.io/j86nf).

**STRENGTHS AND LIMITATIONS OF THIS STUDY**

- This protocol follows the Joanna Briggs Institute methodology and Preferred Reporting Items for Systematic Reviews and Meta-Analyses extension for Scoping Reviews guidelines to ensure a transparent and reproducible approach.
- A comprehensive three-step search strategy across databases and grey literature will maximise study identification and reduce publication bias.
- Independent screening and data extraction by multiple reviewers, with third-reviewer arbitration, will enhance methodological rigour and minimise bias.
- Inclusion of diverse study designs will provide a broad overview of the evidence but may introduce heterogeneity that limits comparability.
- Restricting the review to English-language publications from 2009 onwards, and not assessing intervention effectiveness or study quality, may limit completeness and global generalisability.

## Introduction

 In 2023, over 200 000 adult patients were admitted to intensive care units (ICUs) across England, Wales and Northern Ireland, of whom 82.2% survived to hospital discharge.^1^ However, many survivors experience long-term physical, psychological and social consequences that may persist for up to 5 years post-discharge.^2^ These sequelae contribute to increased healthcare utilisation, reduced employment and substantial socio-economic burden.^3^,^6^ It is estimated that 50–70% of ICU survivors experience post-intensive care syndrome (PICS),^7^ defined as new or worsening impairment in physical, cognitive or mental health that develops during or after an ICU stay and persists beyond hospital discharge.^5 8^ The term *post-critical illness* refers to the period of recovery following resolution of acute organ failure and withdrawal of intensive care therapies, continuing through early rehabilitation in hospital and community settings.^3^

ICU-acquired weakness (ICUAW) is a key contributor to the physical impairments associated with PICS. ICUAW causes diffuse skeletal muscle weakness in approximately 43% of adults requiring critical care.^9 10^ This weakness, which may affect both limb and respiratory muscles, develops during or after an ICU stay and results from complex interactions between systemic inflammation, immobilisation, mechanical ventilation, sedation and underlying illness.^810^,^13^ ICUAW encompasses critical illness polyneuropathy, critical illness myopathy or a combination of both, driven by muscle protein breakdown and impaired neuromuscular function.^9 10^ Clinically, ICUAW typically presents as symmetrical, flaccid limb weakness and is associated with prolonged ventilation, delayed recovery, increased length of stay and higher mortality.^11 14^

Diagnosis often relies on clinical assessment using the Medical Research Council sum score, although electrophysiological testing and muscle biopsy may be used where diagnostic uncertainty persists.^1214^,^16^ For patients, ICUAW can result in profound difficulty performing basic daily activities such as sitting, personal care, feeding or mobilising, and may limit participation long after hospital discharge.^711^,^13 16^ The upper limb is central to self-care, communication, employment and social participation; therefore, upper-limb weakness can significantly constrain independence and quality of life.^9 16 18^ It is defined as reduced strength, coordination or motor control affecting the shoulder, arm, forearm or hand that limits functional activity.^18^

Despite this, rehabilitation research and clinical practice in the ICU have historically prioritised respiratory function and early mobilisation, with comparatively less emphasis on targeted upper-limb interventions.^19 20^ Studies report that up to 59% of ICU survivors experience musculoskeletal pain—particularly in the shoulder—and many show reduced upper-limb strength and health-related quality of life at 6 months post-discharge.^18 21^

There is limited guidance to support healthcare professionals in addressing upper-limb impairment during the acute phase of critical illness. Existing rehabilitation studies primarily focus on whole-body exercise, neuromuscular electrical stimulation, progressive mobility and arm cycling or technology-assisted therapies, although protocols, intensity and clinical reasoning remain inconsistent.^22^,^30^ Delivery appears to be healthcare professional-led, often by physiotherapists or occupational therapists, with limited involvement of family members and little clarity regarding optimal dosage, progression or therapeutic intent.^22 26 27^

Evidence from stroke rehabilitation demonstrates that early, repetitive, task-specific upper-limb therapy supports motor recovery and engagement.^31^,^35^ However, the applicability of post-stroke upper-limb rehabilitation strategies to critically ill populations remains uncertain, given differences in pathology, fatigue tolerance, consciousness and medical stability.

A preliminary search (May 2025) of MEDLINE, the Cochrane Database of Systematic Reviews, and Joanna Briggs Institute (JBI) Evidence Synthesis confirmed no existing scoping or systematic reviews addressing upper-limb rehabilitation for critically ill adults, indicating a clear evidence gap.

### Objective

The objective of this scoping review is to map and summarise the range of upper-limb rehabilitation interventions delivered by healthcare professionals to adults with upper-limb weakness during or following critical illness.

#### Primary review question

“What upper-limb rehabilitation interventions are delivered by healthcare professionals for adults with upper-limb weakness in the ICU?”

#### Review objectives

RO1 To describe the primary clinical ICU diagnosis and/or reasons for upper-limb treatment during and following critical illness in adult patients.

RO2 To explore the clinical indications, contraindications and methods used to identify upper-limb weakness among critically ill adults.

RO3 To identify which outcomes are evaluated and reported in studies of upper-limb rehabilitation in critically ill adults.

RO4 To explore any adverse events attributed to upper-limb rehabilitation in critically ill adults and how these events are defined and monitored.

RO5 To explore perceived barriers and facilitators to implementing upper-limb rehabilitation in adult ICU patients, as described in the existing evidence base.

RO6 To identify which healthcare professionals (eg, occupational therapists, physiotherapists, nurses, rehabilitation assistants and other allied health personnel) deliver upper-limb rehabilitation in the ICU.

RO7 To explore the perceived rehabilitation needs and priorities of patients experiencing upper-limb weakness during intensive care.

Collectively, these objectives aim to inform clinical guidance, optimise intervention planning and highlight areas in which the evidence base is currently limited or absent.

## Methods and analysis

This protocol was developed in alignment with the JBI Manual for Evidence Synthesis and reported using the Systematic Reviews and Meta-Analyses extension for Scoping Reviews (PRISMA-ScR) recommendations. The review is registered on the Open Science Framework (registration number osf.io/j86nf).

### Eligibility criteria

An eligibility framework was developed iteratively to support consistent interpretation among reviewers. Any amendments arising during the review process will be transparently documented in the final report.

### Inclusion criteria

#### Participants

Adult patients aged 16 years or older receiving upper-limb rehabilitation during or following critical illness will be included. The lower age threshold is set at ≥16 years to reflect the population treated in adult ICUs in several health systems (eg, United Kingdom, Australia) and is in line with the UK National Institute of Clinical Excellence (NICE) Guideline CG83.^36^

Patients with progressive neurodegenerative disorders (eg, motor neuron disease, muscular dystrophy) will be excluded due to the differing trajectory of impairment. If studies include mixed populations, data will be extracted only if findings for eligible participants can be distinguished; otherwise, the study will be excluded.

#### Concept

This review will examine the types of upper-limb rehabilitation interventions delivered in the ICU, the timing and intensity of these interventions, the clinical processes, decision-making, outcome measures used and where described patient experience of upper-limb rehabilitation.

Outcome measures will be classified as validated or non-validated, based on evidence cited in the included publications. Data synthesis will involve a descriptive summary for quantitative evidence and thematic analysis for qualitative data. If the timing of intervention (ie, during vs post-ICU admission) cannot be determined, the study will be retained but clearly reported under ‘unclear intervention timing’ in the results.

#### Context

Studies conducted in any country or healthcare system will be considered. The review will focus on upper-limb rehabilitation during the ICU phase or early recovery, without restrictions on sample size. Evidence will be limited to English-language publications from 2009 onwards, reflecting the implementation of NICE Guideline CG83 (2009).^36^ While this may exclude earlier or non-English evidence, it supports methodological feasibility and policy relevance.

#### Types of sources

This review will include experimental and quasi-experimental designs (eg, RCTs, controlled trials, pre–post studies, interrupted time series); Observational studies (eg, cohort, case-control, cross-sectional); Case reports, case series; Qualitative studies (eg, phenomenological, grounded theory, ethnography); Conference abstracts and grey literature where adequate data are reported. Systematic reviews will be screened to identify primary studies; primary data will be prioritised where overlap occurs. Patient societies and online resources have been excluded.

### Search strategy

This review will follow the three-step JBI search strategy.^37 38^

Step 1: An initial, limited search of MEDLINE and CINAHL was conducted to identify relevant keywords and index terms. Step 2: A comprehensive search (online supplemental appendix 1) will be conducted across MEDLINE (Ovid), CINAHL (EBSCO), Embase, Scopus, CENTRAL, PEDro and OpenGrey. A medical librarian will peer-review the search strategy using the PRESS checklist. ^39^Step 3: The reference lists of all included publications will be screened, and forward citation tracking will be performed.

Searches will include publications from March 2009 to August 2025, aligning with the introduction of NICE Guideline CG83.^36^ Language filters will not be applied during database searching; English-language eligibility will be applied during screening.

### Study/source of evidence selection

All citations will be imported into Rayyan for de-duplication. Following a calibration exercise, two independent reviewers (JB and ES) will screen titles and abstracts against the inclusion criteria.

Potentially relevant sources will be retrieved in full, supported through institutional subscriptions and their citation details imported into the JBI System for the Unified Management, Assessment and Review of Information.^40^ These publications will then be assessed by three independent reviewers (JB, ES and ED). Any disagreements will be resolved through discussion or arbitration by the senior reviewer (ED).

Screening will be managed in Rayyan to support blinded conflict handling. The study selection process will be documented using a PRISMA flow diagram.^37 38^

### Data extraction

Data will be extracted by two independent reviewers (JB and ES) using a purpose-designed extraction tool. The tool will be piloted on five studies (JB, ES and ED) and refined as needed. Inter-rater reliability will be assessed using Cohen’s kappa coefficient.

Extracted data will include study metadata (author, year, setting, design, sample characteristics), clinical context of upper-limb treatment (RO1), indications, assessment, scoring and contraindications (RO2), outcome measures and reporting approach (RO3), reported adverse events and monitoring practices (RO4), barriers and facilitators to delivery (RO5), healthcare professional roles (RO6) and patient-reported needs or experiences (RO7).

One reviewer (JB) will contact corresponding authors when clarification is required, with follow-up limited to three contact attempts.

### Data analysis and presentation

Quantitative data will be summarised descriptively (eg, frequencies, ranges, proportions). Where available, effect estimates will be extracted to illustrate patterns across interventions. Qualitative data (eg, interview or focus group findings) will be synthesised using thematic analysis following Braun and Clarke^41^ and guided by JBI guidance for qualitative synthesis.

Findings will be presented via a narrative summary, tabulated mapping of intervention characteristics, thematic summary of perceived needs, experiences, barriers and facilitators.

No assessment of methodological quality or risk of bias will be undertaken, consistent with scoping review methodology.

### Patient and public involvement

A post-ICU Patient Advisory Group (PAG) from a UK NHS centre contributed to the development of the research question and objectives. The PAG will meet twice during the review to advise on interpretation and dissemination planning. Members will receive an induction and INVOLVE guidance pack (National Institute for Health and Care Research public information pack) and will be offered involvement in dissemination outputs.

## Ethics and dissemination

As this review will synthesise previously published data, ethical approval is not required. Findings will be disseminated through peer-reviewed publication, conference presentations, professional networks and open-access repository hosting of extracted datasets where permissible. Outputs will target clinicians, rehabilitation specialists, researchers and policymakers.

## Discussion

This scoping review will comprehensively map the available evidence on upper-limb rehabilitation for critically ill adults, focusing on the type, timing, delivery and professional roles involved in these interventions. By including a wide range of quantitative, qualitative and mixed-methods studies, the review aims to provide a detailed overview of current rehabilitation practices within the intensive care context. This broad inclusion will enable a comprehensive understanding of the scope and diversity of upper-limb rehabilitation approaches but may also introduce heterogeneity that limits the ability to directly compare findings across studies.

The review will not assess the effectiveness of specific interventions or appraise the methodological quality of included studies, consistent with the purpose of scoping reviews. Instead, it will identify key evidence gaps, variations in outcome measurement and inconsistencies in clinical guidance that can inform future research priorities, clinical trials and the development of best-practice recommendations for ICU rehabilitation.

A major strength of this review lies in its adherence to established methodological standards. The protocol follows the JBI methodology for scoping reviews and the PRISMA-ScR reporting framework, ensuring a transparent and reproducible process. The use of a comprehensive three-step search strategy—covering multiple databases and grey literature—will maximise identification of relevant studies and reduce the risk of publication bias. In addition, independent screening and data extraction by multiple reviewers, with arbitration by a third reviewer, will strengthen methodological rigour and minimise bias during study selection and analysis.

The inclusion of diverse study designs, including quantitative, qualitative, mixed-methods, systematic reviews and opinion papers, represents another strength, allowing the synthesis of varied perspectives on upper-limb rehabilitation practice.

Diversity may also contribute to heterogeneity that restricts direct comparison between studies. Restricting the review to English-language publications from 2009 onwards may exclude some relevant evidence and limit global generalisability. Nevertheless, this timeframe aligns with the publication of NICE Guideline CG83 (2009), which marked a pivotal shift in ICU rehabilitation policy and practice, ensuring that findings reflect contemporary clinical frameworks.

Findings from this review will inform the development of evidence-based ICU rehabilitation guidelines and highlight priorities for future research to improve upper-limb recovery in critically ill adults.

## Supplementary material

10.1136/bmjopen-2025-110429 online supplemental file 1
