# Supplementary material for: Upper-limb rehabilitation interventions delivered by healthcare professionals for adult patients in the intensive care unit setting: protocol for a scoping review
Source: BMJ Open. 2025 Dec 3;15(12):e110429. doi: 10.1136/bmjopen-2025-110429 (PMC12682189; doi:10.1136/bmjopen-2025-110429)
Supplement: online supplemental file 1 [file bmjopen-15-12-s001.docx]

**Appendix 1.** Search strategies

**Ovid MEDLINE(R) ALL <1946 to August 20, 2025>**

1 Critical Care/ 64001

2 exp Intensive Care Units/ 118253

3 (critical adj care).ti,ab,kw. 41095

4 (intensive adj care).ti,ab,kw. 219467

5 ICU.ti,ab. 101099

6 CCU.ti,ab. 2249

7 (critical* adj ill*).ti,ab,kw. 78584

8 ventilat*.ti,ab,kw. 223929

9 ICU-acquired.ti,ab,kw. 1343

10 ICUAW.ti,ab,kw. 195

11 or/1-10 529750

12 exp Upper Extremity/ 196649

13 (upper adj (limb* or extremit*)).ti,ab,kw. 68872

14 (forelimb* or forearm* or arm* or axilla or elbow* or hand* or shoulder* or wrist*).ti,ab,kw. 1311095

15 or/12-14 1416131

16 rehab*.ti,ab,kw. 255776

17 exp Rehabilitation/ 382552

18 (physio or physiotherap* or physical therap*).ti,ab,kw. 80109

19 Physical Therapy Modalities/ 42973

20 occupational therap*.ti,ab,kw. 21273

21 (OT or OTs).ti,ab. 25497

22 Occupational Therapy/ 16013

23 occupational therapists/ or physical therapists/ or physical therapist assistants/ 4894

24 or/16-23 632062

25 11 and 15 and 24 1162

26 limit 25 to english language 1038

27 limit 26 to yr="2009 -Current" 784

**Ebsco CINAHL 21/08/2025**

S1 (MH "Critical Care") 27,476

S2 (MH "Intensive Care Units") 50,513

S3 XB critical care 27,421

S4 XB intensive care 84,855

S5 XB ICU 43,731

S6 XB CCU 835

S7 XB critical* N0 ill* 33,867

S8 XB ventilat* 61,793

S9 XB ICU-acquired 633

S10 XB ICUAW 75

S11 S1 OR S2 OR S3 OR S4 OR S5 OR S6 OR S7 OR S8 OR S9 OR S10 197,988

S12 (MH "Upper Extremity") 10,375

S13 XB (upper N0 (limb* or extremit*)) 21,980

S14 XB (forelimb* or forearm* or arm* or axilla or elbow* or hand* or shoulder* or wrist*) 271,523

S15 S12 OR S13 OR S14 285,028

S16 XB rehab* 122,504

S17 (MH "Rehabilitation") 18,471

S18 XB (physio or physiotherap* or physical therap*) 53,764

S19 (MH "Physical Therapy+") 170,901

S20 (MH "Occupational Therapy+") 28,838

S21 (MH "Physical Therapist Assistants") OR (MH "Physical Therapy Assisting") OR (MH "Occupational Therapy Assistants" OR (MH "Occupational Therapy Assisting") ) 1,500

S22 XB (OT or OTs) 6,140

S23 S16 OR S17 OR S18 OR S19 OR S20 OR S21 OR S22 330,469

S24 S11 AND S15 AND S23 530

S25 S11 AND S15 AND S23 [Narrow by Language: - English] 530

S26 S11 AND S15 AND S23 [Limiters - Publication Date: 20090101-20251231] 409
