# Supplementary material for: Upper-limb rehabilitation interventions delivered by healthcare professionals for adult patients in the intensive care unit setting: protocol for a scoping review
Source: BMJ Open. 2025 Dec 3;15(12):e110429. doi: 10.1136/bmjopen-2025-110429 (PMC12682189; doi:10.1136/bmjopen-2025-110429)
Supplement: Supplementary file 2 [file bmjopen-2025-110429.reviewer_comments.pdf]

PEER REVIEW HISTORY

BMJ Open publishes all reviews undertaken for accepted manuscripts. Reviewers are asked to complete a checklist review form and are provided with free text boxes to elaborate on their assessment. These free text comments are reproduced below.

ARTICLE DETAILS

Title (Provisional)

Upper-limb rehabilitation interventions delivered by healthcare professionals for adult patients in the intensive care unit setting: protocol for a scoping review

Authors

Bruce, James Matthew; Swingwood, Ema; Barrett, Sarah; Dures, Emma

---

VERSION 1 - REVIEW

**Reviewer**	**1**
**Name**	**Nydahl, Peter**
**Affiliation**	**University Hospital of Schleswig-Holstein, Nursing Research**
**Date**	**01-Oct-2025**
**COI**	**None**

---

thank you very much for the opportunity to review the submitted manuscript. The manuscript includes a study protocol about a scoping review of interventions for rehab of upper limbs in critically ill patients.

SERIOUS CONCERNS

Line 30: section abstract appears to be wordy. I suggest to reduce section introduction to 3 or 4 sentences.

Line 44: please add your methods for analysing the studies.

Section introduction: I am missing a sentence starting with “Upper limb weakness is defined as …” (references). Please add your core definition/s to the introduction. You may add a definition of “health care workers”, including OT, PT, RN … anyone else, or not? What if families assist?

Section introduction: and I am missing a sentence “post critical illness is defined as …” otherwise I am confused where the rehab takes place and what type of HCW are involved.

L172 why 16 years? This is rather unusual, instead of 18 years. Please explain.

L179: HCW in ICU? But the treatment should be post critical illness. I do not understand this, please explain or revise. See also L184 “during ICU admission”, which is a procedure taking one hour to welcome a patient on ICU and start treatment. Again, report clearly the timing of the intervention and involved clinicians (trained ICU PTs are different from PTs in rehab facilities – both are excellent, but not exchangeable). In some parts, rehab is targeted on ICU, in other parts it is post ICU. This is is confusing … please check your whole manuscript and revise

L263: please add more information to your analysis. This is important. Report in one paragraph the planned analysis of quantitative data including statistics, and in a second one the summary of qualitative data.

MINOR CONCERNS

L74: here you mention “grey literature” which is not reported in section abstract. Please revise.

L132: to be consistent with your subject (ICU), I suggest add a sentence such as “But this evidence is limited to patients after stroke, and the transfer to general ICU patients appears to be questionable”, or else.

L138: please add the date (years, month)

L142: if you include “to describe an overview of upper limb weakness …” you have to report prevalence/incidence, outcome and else. But you focus on rehab, hence the aim should be “to describe an overview of interventions for rehabilitation of critically ill patients with upper limb weakness”, or else.

L194 what about conference’ posters? Later it is mention in line 224. Revise.

L196 please explain reasons for action research and feminist research, since the connection is not obvious.

By the way: you do not include patient reports, patients’ societies (e.g. ICUsteps) and no online resources such as websites, discussion boards, or apps? If not, just report with an explanation.

L210 always add PEDRo for rehab interventions.

L220 have a peer- review with a medical librarian for improving your search strategy – it upgrades the quality of your search!

L225: if a study / report is online, it is published. With your search strategy, you cannot identify “unpublished” papers.

L230: you are mentioning “studies” but according to the inclusion criteria, you include “papers” too. Hence, it should be “studies and papers”, or simple “publications”, or else. Please revise.

L238: by the way: who pays the bill for paying full texts?

Add a section “limitations” and what you do to minimise a potential bias, or why it is neglectable (in this case with references)

Check and update your literature. In general, included references should not be older than 5 years, except these are very important or milestones studies, such as the NICE guideline. Of course, the results of your search can be different.

Very interesting work! I am looking forward to your revision and later, for your results!

**Reviewer**	**2**
**Name**	**Simões, João Lindo**
**Affiliation**	**University of Aveiro, School of Health Sciences (ESSUA)**
**Date**	**09-Oct-2025**
**COI**	**None**

Thank you for the opportunity to read your work. Below is some feedback intended to help you to strengthen the manuscript.

General Comments

This manuscript presents a well-developed and timely protocol for a scoping review on upper limb rehabilitation interventions for adult patients in the intensive care unit. The topic is highly relevant given the increasing recognition of post-intensive care syndrome and ICU-acquired weakness, and the relative lack of targeted rehabilitation strategies for the upper limb in critically ill populations. The manuscript demonstrates methodological rigour by adhering to Joanna Briggs Institute (JBI) and PRISMA-ScR guidelines, ensuring transparency and reproducibility. The inclusion of a broad range of study designs and the incorporation of Patient and Public Involvement (PPI) are important strengths that will enhance the breadth and applicability of the review.

Nevertheless, there are several areas where the manuscript would benefit from refinement. The introduction and abstract could be made more concise to avoid redundancy and to more sharply emphasise the research gap. The objectives, while comprehensive, partially overlap and could be refined for clarity. The methods section, although robust, would benefit from additional details on how heterogeneity across study designs will be addressed, as well as how qualitative findings will be synthesised. Furthermore, the limitations of language and date restrictions should be more explicitly acknowledged. Finally, the dissemination strategy could be expanded to demonstrate more clearly how the findings will be translated into practice and policy. With these adjustments, the manuscript would be well positioned to make a meaningful contribution to the literature on ICU rehabilitation.

Specific Comments:

Title and Abstract

The title is clear, precise, and accurately reflects the focus of the study. The abstract is generally well structured and comprehensive. However, it is somewhat lengthy and could be streamlined to improve clarity, especially in the background section. The research gap should be stated more explicitly at the outset of the abstract to better highlight the novelty of the study.

Introduction

The introduction provides a strong rationale and convincingly frames ICU-acquired weakness and post-intensive care syndrome as significant clinical problems. It also effectively situates the study in relation to the literature on stroke rehabilitation, which is a thoughtful comparison. That said, some sections are repetitive, particularly regarding prevalence figures and the impact of ICUAW, and would benefit from being condensed. The extrapolation from stroke rehabilitation to ICU settings is promising but should also be more critically discussed, acknowledging differences in populations and care contexts. The claim that no reviews exist on this topic should be substantiated more firmly by explicitly presenting the findings of the preliminary search.

Objectives

The objectives are comprehensive and systematically address different aspects of rehabilitation, from clinical diagnosis to patient experience and healthcare worker involvement. However, some objectives overlap conceptually, such as SO2 (indications and contraindications) and SO3 (identification of weakness). Refining or merging these could improve clarity and prevent redundancy. Furthermore, it would strengthen the protocol to explicitly link each objective to expected outputs, such as informing guideline development or clinical practice recommendations.

Methods

The methods are described in detail and appropriately follow established scoping review frameworks. The three-step search strategy is well designed, and the appendices provide transparency. Nevertheless, the restriction to English-language publications introduces language bias, which should be more explicitly acknowledged and discussed. While the rationale for the 2009 cut-off is understandable (linked to NICE guidelines), further justification would improve transparency, and it may be valuable to note that older literature could still provide useful historical context. More importantly, the methods could benefit from greater detail on how the review will manage heterogeneity across different study designs. For example, will the team stratify results by study type or intervention type? For qualitative data, more detail on the planned synthesis approach (e.g., thematic or framework analysis) would add clarity.

Data Extraction and Analysis

The use of independent reviewers and piloting of the extraction tool are commendable and demonstrate methodological rigour. However, it would be helpful to specify whether inter-rater reliability will be formally assessed (for example, using a kappa statistic). Additionally, while the planned narrative, tabular, and graphical presentations are appropriate, the manuscript should clarify how qualitative findings will be systematically synthesised.

Ethics and Dissemination

The ethics statement is accurate for this type of study, and the plan to use open repositories for data sharing aligns well with principles of transparency and reproducibility. However, the dissemination section could be more developed. The authors should specify their target audiences (clinicians, rehabilitation specialists, researchers, policy-makers) and describe tailored dissemination strategies for each. It would also be valuable to discuss how the findings might inform clinical guidelines or be translated into recommendations for practice.

Discussion

The discussion reiterates the importance of the topic and highlights key gaps in the current evidence base. While this section is coherent, it largely repeats background material rather than providing a forward-looking perspective. It could be strengthened by addressing how the findings of this review may shape clinical practice, guideline development, or future research priorities. The authors should also acknowledge anticipated challenges, such as managing heterogeneous interventions and outcome measures.

References

The references are current and include key sources relevant to ICU rehabilitation and scoping review methodology. However, the reference list is heavily weighted towards UK and European sources. Incorporating more international studies would enhance the global relevance of the protocol. In addition, greater detail on the preliminary search results would substantiate the novelty claim.

Overall Recommendation

This is a promising and methodologically sound protocol that addresses a clinically important and underexplored area of rehabilitation research. The manuscript would be strengthened by streamlining redundant sections, refining the objectives, providing more detail on handling heterogeneity and qualitative synthesis, explicitly acknowledging limitations of language and date restrictions, and expanding the dissemination strategy. Addressing these points will enhance the clarity, rigour, and impact of the protocol, ensuring that it makes a valuable contribution to the ICU rehabilitation literature.

**Reviewer**	**3**
**Name**	**Gutiérrez-Arias, Ruvistay**
**Affiliation**	**Instituto Nacional del Tórax**

**Date**	**12-Oct-2025**
**COI**	**None**

Please find below my comments and suggestions regarding your manuscript. I hope you find them useful:

Title:

- It is appropriate.

Abstract:

- The PRISMA-ScR statement provides recommendations for reporting a ScR transparently and comprehensively. This is relatively clear in lines 45-46, but not in lines 58-59. Please make the necessary edits.

- Please report the databases you will consult in the second stage of your search strategy.

- Will you use any tools (AI-based or otherwise) to facilitate the conduct of your ScR? For example, Rayyan, TERAL tools, or others. Please report this.

- In the strengths and limitations section, you mention that you will include grey literature. Please add this information to the abstract.

Article Summary:

- If you recognise that restricting the publications included to English and from 2009 onwards is a limitation of your study (with which I agree), why not change these criteria? You are still in the publication phase of your protocol.

Introduction:

- Edit the citations: For example, (3,4,5,6) should be (3-6).

- I suggest editing the following idea: 'The physical manifestation of PICS is known as ICU-acquired weakness (ICUAW)'. 'ICUAW leads to physical alterations of PICS' better reflects what you are trying to communicate.

- I suggest deleting the paragraph between lines 102 and 105, as it strays from the central idea of the protocol.

- This section is well written.

Methods and analysis:

- I suggest reporting objectives SO1 to SO8 together with the main question at the end of the introduction. Report the 'secondary' objectives as secondary questions, as recommended in the JBI ScR manual.

- In the first paragraph of the methods section, report the methodological guide you used to structure the protocol and plan the conduct of the ScR. In addition, state the protocol you used for this ScR. And report the protocol registration number in OSF.

- Inclusion criteria; Participants: What will you do if a study includes participants with and without neurological disorders?

- Inclusion criteria, Concept: While you will include validated and unvalidated outcome measures, how will you determine whether an outcome measure is validated?

- Inclusion criteria, Context: What will you do if a study follows participants throughout their hospital stay, and it is not possible to clearly distinguish when upper limb rehabilitation began?

- Inclusion criteria, Context: The date and language of the studies should be reported in the 'Types of resources'. In addition, explain the reason for limiting your search to 2009 onwards. The reason you give is unclear. Also, explain the reason for restricting the inclusion of studies to the English language.

- Inclusion criteria, Type of resources: It does not make sense to include secondary studies in your ScR. Will you address and resolve the overlap of primary studies if you identify a systematic review that meets the inclusion criteria? Also, what do you mean by 'depending on the research question'? Please be specific.

- Review the three steps of your search strategy. While it is in line with the JBI manual, it repeats information and confuses step 1 with step 2. In addition, steps 2 and 3 contain similar information.

- The information on the publication date and language limits has already been presented in the eligibility criteria. Present this information only in the eligibility criteria, and in the search strategy report whether you limited your syntax with publication date and language filters.

- Report all the databases in which you will search for studies.

- Report the abbreviation SUMARI in line 240.

Discussion:

- This section looks fine.

VERSION 1 - AUTHOR RESPONSE

Reviewer Comment	Author Response	Line
Update the 'Methods and analysis' section to ensure that it contains an adequate description of the search strategy and selection criteria, including all databases, date ranges, language	We thank the reviewer for this detailed comment. The 'Methods and analysis' section has been expanded to describe all databases, date range, inclusion and	Lines<br><br>35-47

Reviewer Comment	Author Response	Line
restrictions, and key inclusion/exclusion criteria. Ensure consistency between abstract and main text.	exclusion criteria, and language parameters. These updates are consistent across the abstract and main text.	152 - 236
Please change the heading 'Ethical and Safety Considerations, Dissemination Plan' to 'Ethics and dissemination'.	Thank you for noting this. The heading has been revised to 'Ethics and dissemination'.	Line 248
Move the 'Patient and Public Involvement' statement to immediately precede the 'Ethics and dissemination' section.	We appreciate this suggestion. The section has been relocated as recommended.	Line 241
Ensure that the Discussion section fully addresses methodological limitations, including those highlighted in the 'Strengths and limitations' section.	We agree this strengthens transparency. The Discussion now includes a comprehensive account of methodological limitations.	Lines 254 - 288
Clarify the meaning and timing of Appendix 1 search results and whether the search will be repeated.	We thank the reviewer for raising this. Clarification has been added specifying that Appendix 1 presents search dates for the current step, which will be updated if the search is repeated.	199-201
Appendix 2 appears missing; please check and upload separately as 'Supplemental Material'.	Appendix 2 contained the data extraction tool and has now been removed from the main manuscript. It is not required as separate supplementary material.	—
L30: The abstract appears wordy. Please reduce the introduction to three or four sentences.	We appreciate this feedback. The abstract introduction has been condensed to three concise sentences to improve readability.	Lines 26-33
L44: Please add your methods for analysing the studies.	The abstract has been updated to include a clear summary of analytical methods.	Lines 34-47
Add a definition for "upper limb weakness" with supporting references.	A definition of upper limb weakness and an accompanying reference have been added to the introduction.	Lines 105-106
Add definitions for key terms such as "health care professional" and describe family contributions.	Definitions for 'health care professional' and 'family contribution' have been incorporated for clarity.	Lines 116 - 118

**Reviewer Comment**	**Author Response**	**Line**
Include a definition for “post-critical illness” to clarify where rehabilitation occurs and who is involved.	A definition of ‘post-critical illness’ and an American reference have been added.	Lines 85-87
Justify why participants aged ≥16 years were selected rather than ≥18 years.	The age criterion is now justified in the text and aligned with NICE 83 clinical guidelines.	Lines 162-165
Clarify whether the focus is ICU or post-ICU rehabilitation.	We agree that this required clearer framing. The manuscript has been revised throughout to focus on early, acute ICU rehabilitation.	—
Add more detail on planned analysis, distinguishing quantitative and qualitative approaches.	The analysis section has been expanded to describe quantitative and qualitative analysis plans in separate paragraphs.	Lines 231-240
Mention of “grey literature” in the abstract should align with the main text.	The abstract now includes grey literature as specified in the main methods.	Line 45<br>200
To align with ICU focus, note that prior evidence mainly concerns stroke patients and may not generalize.	A sentence acknowledging limited transferability from stroke to ICU populations has been added.	Lines 120-122
Add the date (year/month) for the search.	The timeline of the database search has been specified.	40<br>205
Who pays for the full publications.	A sentence has been added supported through institutional subscriptions.	212.
Clarify the aim statement to focus on “interventions for rehabilitation” rather than an overview of weakness.	The aim has been reworded to emphasise rehabilitation interventions for critically ill patients with upper limb weakness.	131-132
Include conference posters and ensure consistent reporting throughout.	References to conference abstracts and posters have been harmonized across the manuscript.	-.
Explain rationale for action and feminist research approaches.	Thank you for highlighting this. This was originally linked to JBI’s inclusivity of diverse paradigms but agree it does not fit	-

**Reviewer Comment**	**Author Response**	**Line**
	well with this article, so it has been removed.	
Explain why patient organizations or online resources are excluded, if applicable.	A statement clarifying the exclusion of patient societies and online resources has been added.	Line 193-194
Always include PEDro in rehabilitation searches.	PEDro has been added to the search strategy.	Line 200
Consider peer review of search strategy by a medical librarian.	Collaboration with a medical librarian is planned; this has been noted and referenced.	Lines 200-201
Clarify use of the term “unpublished papers.”	We agree with the reviewer. The term has been removed for accuracy.	-
Replace “studies” with “publications” for consistency.	The term “publications” is now used consistently throughout the manuscript.	-
Indicate how access to full-text papers will be covered.	A statement regarding access to full texts has been added.	Lines 212 - 214
Add a limitations section and describe how bias will be minimized.	A dedicated limitations subsection has been added to the Discussion, aligned with PRISMA-ScR guidelines.	Lines 282-288
Update references so that most are within the last five years, retaining only essential older works.	The reference list has been updated to include recent and relevant literature	—

**Reviewer Comment**	**Author Response**	**Line**
The abstract should be streamlined for clarity, particularly in the background section. The research gap should be stated more explicitly to highlight the novelty of the study.	We thank the reviewer for this helpful feedback. The abstract has been revised for conciseness, and the research gap and study novelty are now clearly articulated at the outset.	Lines 27-33

Reviewer Comment	Author Response	Line
Clarify differences between stroke rehabilitation and ICU populations.	Acknowledging this important distinction, we have added a statement noting the uncertain generalisability of stroke rehabilitation findings to ICU populations.	Lines 118-120
The claim that no reviews exist on this topic should be substantiated more firmly by presenting results of the preliminary search.	We appreciate this suggestion. The manuscript now explicitly reports the preliminary search findings to support our claim of novelty.	Lines 110-116<br>121-123
Objectives SO2 (indications/contraindications) and SO3 (identification of weakness) could be refined or merged to avoid redundancy.	Thank you for this valuable comment. The objectives have been merged and refined for improved clarity.	Lines 132-133
Explicitly link each objective to expected outputs, such as informing guideline development or clinical practice.	We have revised the conclusion to clearly connect each objective with anticipated outputs, including contributions to guideline development and clinical practice.	Lines 147-148
Restricting to English-language publications may introduce language bias; this should be explicitly acknowledged.	We agree and have added a discussion of potential language bias in both the methods and limitations sections.	Lines 183-184<br>203-204<br>276-277
Further justify the 2009 start date; the rationale linked to NICE guidelines could be made clearer.	We have clarified that 2009 marks a major policy shift in ICU rehabilitation practice, supporting our choice of start date.	Lines<br>202 - 203<br>278-279
Provide greater detail on managing heterogeneity across study designs.	Additional text has been included to describe strategies for handling methodological heterogeneity across included studies.	250-254

Reviewer Comment	Author Response	Line
Confirm that inter-rater reliability will be assessed and specify how.	We have now specified that inter-rater reliability will be statistically assessed and reported.	Lines 216-218
The dissemination section should be more developed.	The dissemination plan has been expanded to include details of academic, clinical, and public dissemination strategies.	Lines 242-246
Strengthen the Discussion by outlining how findings may influence clinical practice, guideline development, and future research, and by acknowledging challenges such as heterogeneous interventions and outcomes.	We thank the reviewer for this excellent point. The Discussion has been rewritten to address these aspects comprehensively.	Lines 248-279

Reviewer Comment	Author Response	Line
Amend the abstract to include the PRISMA-ScR statement.	We thank the reviewer for this observation. The abstract has been updated to include reference to the PRISMA-ScR framework.	Lines 35-37
Report the databases that will be consulted in the second stage of the search strategy.	This has been addressed by listing all databases in the abstract and methods sections.	Lines 196-198
Indicate whether any AI-based or other tools (e.g., Rayyan, TERAL) will be used to facilitate the ScR process.	We appreciate this suggestion. We have added details specifying that such tools will be used to support the review process.	Lines 47-48
The inclusion of grey literature in the strengths and limitations section should also be reflected in the abstract.	We thank the reviewer. Grey literature inclusion has now been noted in the abstract for consistency.	Lines 46 -47
Limiting publications to English and from 2009 onward may restrict findings; why not broaden criteria?	We acknowledge this limitation but have retained these criteria due to time constraints for translation and the 2009 practice shift. This limitation is discussed in the manuscript.	Lines 182 - 184
Edit citations (e.g., use 3–6 instead of 3,4,5,6).	We thank the reviewer. All in-text citations have been reformatted as suggested.	—

Reviewer Comment	Author Response	Line
Revise the sentence: "The physical manifestation of PICS is known as ICU-acquired weakness (ICUAW)" to better reflect causation.	We agree. This has been revised to: ICU-acquired weakness (ICUAW) is a key contributor to the physical impairments associated with PICS	Line 87 -88
Delete clinical assessment part of the introduction	Thank you for your thoughts on this but we feel it's important for the reader to understand how diagnosis of ICUAW is completed and the ongoing uncertainly.	Line 96-98
Report the abbreviation SUMARI	Thank you for highlighting this, we have written in full and added the abbreviation.	Line 210
Review the three steps of your search strategy. While it is in line with the JBI manual, it repeats information and confuses step 1 with step 2. In addition, steps 2 and 3 contain similar information.	Thank you for highlighting the lack of clarity. We have streamlined the text and removed the repeated wording.	Line 193-200

VERSION 2 - REVIEW

**Reviewer**	**3**
**Name**	**Gutiérrez-Arias, Ruvistay**
**Affiliation**	**Instituto Nacional del Tórax**
**Date**	**14-Nov-2025**
**COI**	

The authors have adequately addressed my comments and suggestions.
